# Multiple Bowen's disease on the finger associated with human papillomavirus type 34

**DOI:** 10.1002/ski2.238

**Published:** 2023-05-01

**Authors:** Satoru Yonekura, Gyohei Egawa, Takaya Komori, Kenji Kabashima

**Affiliations:** ^1^ Department of Dermatology Kyoto University Graduate School of Medicine Kyoto Japan

## Abstract

Human papillomavirus (HPV) infection has been suggested as a potential risk factor for Bowen's disease. Here, we report a case of a 40‐year‐old man with Bowen's disease on the finger showing a discontinuous skip lesion, in which HPV‐34 was detected. Our case is a reminder that the possibility of multiple lesions must be considered when Bowen's disease occurs on the finger.

## Graphical abstract

We report a case of Bowen's disease on the finger showing a discontinuous skip lesion, in which HPV‐34 was detected. Our report shows the infectious aspect of Bowen's disease, especially when it appears on the finger. This is especially important during lesion excision to prevent the oversight of multiple lesions and secondary HPV infections around the surgical site.

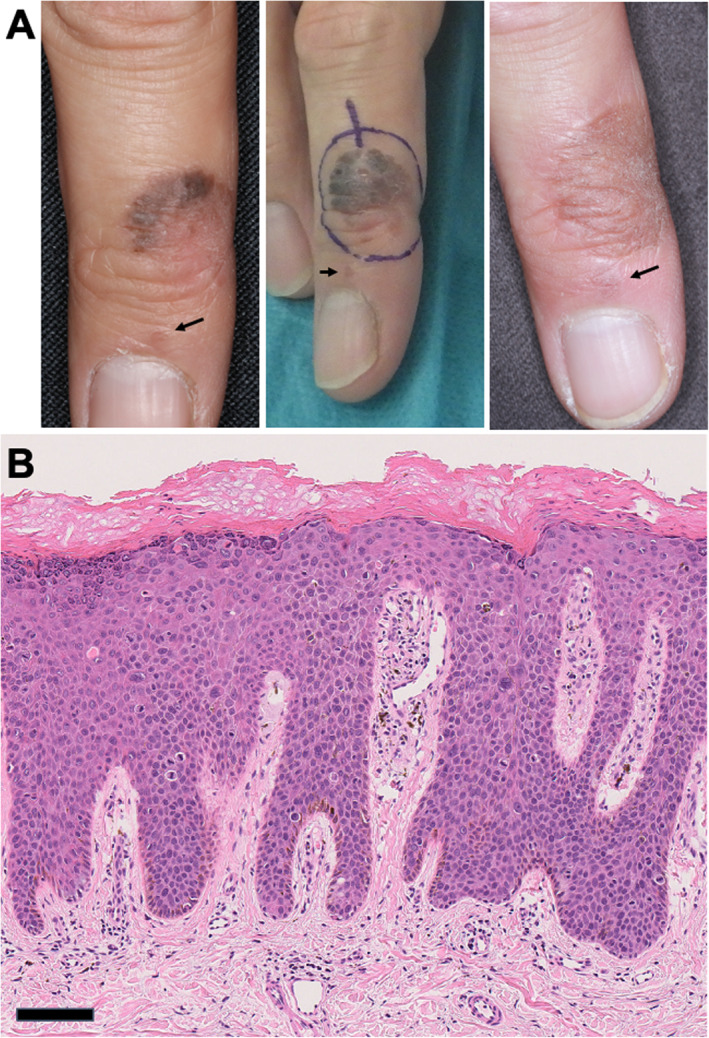

## 1 INTRODUCTION

Human papillomavirus (HPV) infection has been suggested as a potential risk factor for Bowen's disease, a cutaneous squamous cell carcinoma (SCC) in situ.^1^ HPV type is classified into five genera (α, β, γ, μ, and ν), and mucosal high‐risk α‐HPV type, such as HPV‐16, has been most highly detected in the lesion.^1^, ^2^ Here, we report a case of Bowen's disease on the finger with a discontinuous skip lesion, in which HPV‐34 was detected from both lesions.

## 2 CASE REPORT

**FIGURE 1:**
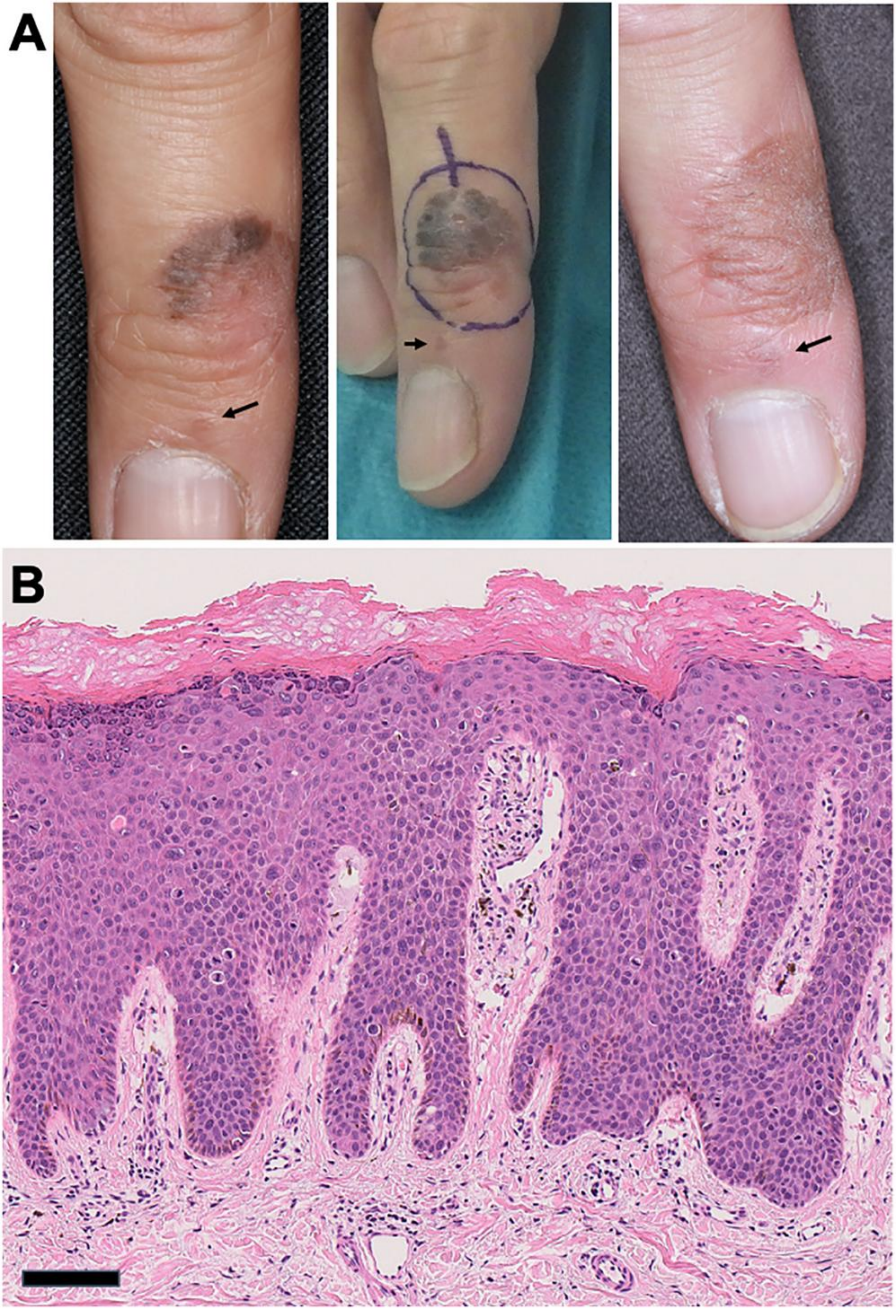
Bowen's disease on the dorsal side of the right middle finger. (a) Clinical manifestation on the initial examination (left), excisional design (middle), and the manifestation 1 year after the first excision (right). Arrows represent the periungual lesions of Bowen's disease. (b) The histology shows that atypical basaloid cells replaced the full thickness of the epidermis with some clumping cells and individual cell keratinisation (haematoxylin and eosin staining, scale bar: 100 μm).

A 40‐year‐old man visited our hospital complaining of brown plaques on the dorsal side of the right middle finger (Figure 1a, left). The plaque was round, flat, well‐demarcated, and 12 mm in diameter. The clinical differential diagnosis includes seborrhoeic keratosis, wart, nevus, psoriasis, pigmented actinic keratosis, and basal cell carcinoma.^3^ Skin biopsy revealed that atypical basaloid cells replaced the full thickness of the epidermis, and some clumping cells and individual cell keratinization were observed (Figure 1b), suggesting the diagnosis of Bowen's disease. He had no history of HPV‐associated tumours in the anogenital area. We excised the lesion with a 3 mm margin and covered it with skin grafting (Figure 1a, middle). The surgical margin was negative. We observed the lesion every 3 months after the surgery. One year later, we noticed that brown plaque had enlarged on the periungual area of the right middle finger (Figure 1a, right). We shaved the lesion, and the histological examination revealed that this lesion was also consistent with Bowen's disease. A polymerase chain reaction was performed with the consensus primers MY09/MY11, which amplifies a large number of HPV types from the L1 region, and HPV‐34 was detected from both the first and second excised lesions.

## 3 DISCUSSION

The noteworthy feature of this case is the discontinuous spread of multiple Bowen's disease lesions on the same finger amidst normal skin. HPV‐34 was identified in both lesions, indicating the possibility of an HPV infection following minor trauma as the cause of the periungual skip lesion. This case serves as a valuable reminder of the infectious nature of Bowen's disease.

Approximately 30% of Bowen's disease cases are associated with HPV, with a higher incidence observed in cases affecting the finger, nail, and genital area.^4^ The most common HPV subtype found in ungual and periungual Bowen's disease/SCC is HPV‐16, and most of the other HPV subtypes detected in these Bowen's disease belong to high‐risk α‐HPV oncogenic clade.^1^ HPV‐34 is also classified as α‐HPV^5^ and was first identified from Bowen's disease on the finger^6^ as seen in our case. A previous study has shown that the expression levels of Ki‐67 and p16^INK4a^, cell proliferation markers, are significantly higher in α‐HPV‐associated digital Bowen's disease/SCC as compared with HPV‐negative tumours, suggesting the presence of high‐risk α‐HPV in digital Bowen's disease/SCC is associated with high local invasiveness.^7^ Therefore, Bowen's disease on the finger should be carefully evaluated for the possibility of multiple lesions and high local invasiveness.

The potential for HPV to spread is especially relevant in digital Bowen's disease. In our case, we initially overlooked the small periungual skip lesion, which can be observed retrospectively in the pictures (Figure 1a, arrows). A literature review conducted by Riddel et al. indicated that multiple cases of digital Bowen's disease are typically found on different fingers,^1^ making our case unique. The peri‐ and/or subungual lesions of the distal digits are the primary sites of HPV‐associated Bowen's disease/SCC outside the genital area,^8^ likely due to the fingertips' susceptibility to minor trauma and frequent contact with the genital area. As such, when Bowen's disease is observed on the fingers, examination of not only the patient's genital lesions but also those of their sexual partner is recommended.^1^

In conclusion, we should remind the infectious aspect of Bowen's disease, especially when it appears on the finger. This consideration is especially important during lesion excision to prevent the oversight of multiple lesions and secondary HPV infections around the surgical site.

## CONFLICT OF INTEREST STATEMENT

None to declare.

## AUTHOR CONTRIBUTIONS

**Satoru Yonekura**: Data curation (equal); investigation (equal); writing – original draft (lead). **Gyohei Egawa**: Conceptualization (equal); data curation (equal); formal analysis (equal); investigation (equal); methodology (equal); supervision (equal); writing – review & editing (equal). **Takaya Komori**: Data curation (equal); investigation (equal); writing – review & editing (supporting). **Kenji Kabashima**: Supervision (supporting); validation (supporting); writing – review & editing (supporting).

## ETHICS STATEMENT

The activity of reporting a case does not have to be reviewed by an IRB, which does not meet the definition of ‘research’, which is: ‘a systematic investigation, including research development, testing and evaluation, designed to develop or contribute to generalizable knowledge’. The patient in this manuscript have given written informed consent to publish his case details.

## Data Availability

Data sharing not applicable—no new data generated.
